# Molecular mechanism of ferulic acid and its derivatives in tumor progression

**DOI:** 10.1007/s43440-023-00494-0

**Published:** 2023-05-18

**Authors:** Xingxun Bao, Wei Li, Ruixue Jia, Dandan Meng, Hairong Zhang, Lei Xia

**Affiliations:** 1 School of Chinese Medicine, Shandong University of Traditional Chinese Medicine, Jinan, 250355 People’s Republic of China; 2 Department of Obstetrics and Gynecology, Linyi Third People’s Hospital, Linyi, People’s Republic of China; 3 Department of Obstetrics and Gynecology, Shandong Provincial Third Hospital, Jinan, 250031 People’s Republic of China; 4 Department of Pathology, Shandong University of Traditional Chinese Medicine, Jinan, 250355 People’s Republic of China

**Keywords:** Ferulic acid, Tumor, Derivative, Nanoliposome, Mechanism of action

## Abstract

Cancer is a significant disease that poses a major threat to human health. The main therapeutic methods for cancer include traditional surgery, radiotherapy, chemotherapy, and new therapeutic methods such as targeted therapy and immunotherapy, which have been developed rapidly in recent years. Recently, the tumor antitumor effects of the active ingredients of natural plants have attracted extensive attention. Ferulic acid (FA), (3-methoxy-4-hydroxyl cinnamic), with the molecular formula is C_10_H_10_O_4_, is a phenolic organic compound found in ferulic, angelica, jujube kernel, and other Chinese medicinal plants but is also, abundant in rice bran, wheat bran, and other food raw materials. FA has anti-inflammatory, analgesic, anti-radiation, and immune-enhancing effects and also shows anticancer activity, as it can inhibit the occurrence and development of various malignant tumors, such as liver cancer, lung cancer, colon cancer, and breast cancer. FA can cause mitochondrial apoptosis by inducing the generation of intracellular reactive oxygen species (ROS). FA can also interfere with the cell cycle of cancer cells, arrest most cancer cells in G_0_/G_1_ phase, and exert an antitumor effect by inducing autophagy; inhibiting cell migration, invasion, and angiogenesis; and synergistically improving the efficacy of chemotherapy drugs and reducing adverse reactions. FA acts on a series of intracellular and extracellular targets and is involved in the regulation of tumor cell signaling pathways, including the phosphatidylinositol 3 kinase (PI3K)/protein kinase B (AKT), B-cell lymphoma-2 (Bcl-2), and tumor protein 53 (P53) pathways and other signaling pathways. In addition, FA derivatives and nanoliposomes, as platforms for drug delivery, have an important regulatory effect on tumor resistance. This paper reviews the effects and mechanisms of antitumor therapies to provide new theoretical support and insight for clinical antitumor therapy.

## Introduction

Malignant tumors are a multifactor and multistep disease, the incidence of which has increased in recent years [1], but the pathogenesis has not been fully elucidated, so there is no effective aetiological treatment. In 2023, 1,958,310 new cancer cases and 609,820 cancer deaths are projected to occur in the United States [2]. Globally, there will be an estimated 28.4 million new cancer cases by 2040, a 47 percent increase over the number of new cases in 2020 [3]. This trend reflects the growing burden that cancer imposes on global health systems and highlights the need for continued efforts to prevent, detect, and treat this disease.

Current cancer treatments include surgical intervention, radiation, and chemotherapy drugs, which often kill healthy cells and cause a host of adverse reactions in patients. With the continuous development of medicine, new therapies such as induction chemotherapy and targeted therapy have been applied to cancer treatment. Compared with traditional chemoradiotherapy, the survival time of patients has been significantly extended with these new therapies, but the adverse reactions and quality of life have not been fundamentally improved [4, 5]. Therefore, the continuous exploration of safe and efficient antitumor treatments is still the main research direction of contemporary oncology. Natural plant components play a significant role in the prevention and treatment of cancer, have significant efficacy in improving the clinical symptoms, quality of life, and prognosis of cancer patients, and have gradually become an important means of cancer prevention and treatment.

Ferulic acid (FA), a phenolic substance widely found in plant cell walls [6–8], is an important active component of many traditional Chinese medicines. It has stable physical and chemical properties, outstanding pharmacological activity, and few toxic and side effects on the human body. Its pharmacological activities mainly include antioxidant [9–11], anti-inflammatory [12, 13], anti-diabetic [14, 15], blood pressure-lowering [16], hepatoprotective [17, 18], and immunoregulatory [19, 20] activities. With the deepening of pharmacological research on FA, it has shown significant antitumor biological activity [21–24] and is expected to become a potential drug for the treatment of malignant tumors. In this paper, progress related to the antitumor mechanism of FA was reviewed to provide new ideas for solving the current problems of poor efficacy, high toxicity, and drug resistance of traditional antitumor drugs.

## Induction of tumor cell apoptosis after FA intervention

Tumor cells can grow without limit and resist programmed death caused by genes. This malignant behavior of tumors not only increases the difficulty of treatment but is also an important cause of cancer-related death. Apoptosis is the most important form of programmed cell death [25]. FA has a significant effect on inducing apoptosis.

### Tumor protein 53 (P53)

The expression of p53, a key tumor suppressor gene [26], is altered in most cancers. The loss of p53 function is often a prerequisite for the development of cancer [27]. Niu et al. [28] showed that FA could induce the apoptosis of gastric cancer SDC-7901 cells, and the mechanism involved FA-mediated upregulation of the mRNA and protein expression of p53. Umut et al. [29] observed that FA could increase the expression level of p53 in MIA PaCa-2 pancreatic cancer cells while reducing the expression levels of cyclin D1 and cyclin-dependent kinase (CDK) 4/6. In addition, FA was found to reduce colony formation and inhibit cell invasion and migration. The results suggested that FA could promote the apoptosis of MIA PaCa-2 cells by increasing the expression of p53, thus showing an antitumor effect. Folate-mediated metabolism is crucial to the stability and function of the genome and affects the occurrence and development of tumors [30]. Kumar et al. [31] evaluated the targeted efficacy of chitosan-coated FA-loaded solid lipid nanoparticles and folate conjugate (FFA) in colon cancer. Compared with the control cells, FFA-treated HT-29 cells showed increased p53 levels, increased apoptosis, and loss of mitochondrial membrane potential. Further studies showed that FFA could trigger the release of cytochrome C in colon cancer cells, and the expression of cysteinyl aspartate-specific proteinase (caspase)-9 and -3 increased after FFA treatment. Together, these results indicate that FFA can activate p53-mediated intrinsic apoptosis, suggesting that these targeted biomaterials could be used as an effective drug in cancer therapy.

### B-cell lymphoma-2 (Bcl-2) protein family

Bcl-2 plays an important role in controlling cell apoptosis and enhancing cell survival. miR-34 is abnormally expressed in the tumor process and is considered a tumor suppressor microRNA due to its synergistic effect with the tumor suppressor gene p53 [32]. Increasing the expression of miR-34a can inhibit Bcl-2 and increase apoptosis [33]. In a human cervical cancer xenograft model, FA treatment was found to reduce tumor weight in a dose-dependent manner, increase miR-34a expression, downregulate Bcl-2 protein expression, and upregulate caspase-3 protein expression [34]. Therefore, the inhibitory effect of FA on the growth of transplanted tumors of human cervical cancer in nude mice may be realized by upregulating miR-34a, thus inhibiting the expression of its target gene Bcl-2, initiating the apoptotic pathway and promoting cell apoptosis. Zhang et al. [35] used FA treatment on gastric cancer MGC-803 cells, and the results showed that FA could upregulate the expression of Bcl-2-associated X (Bax) mRNA and protein and downregulate the expression of Bcl-2 mRNA and protein, thus effectively inducing the apoptosis of MGC-803 cells and inhibiting their proliferation. These results suggest that the mechanism of FA-induced apoptosis may be related to the activation of the endogenous mitochondrial apoptosis pathway. Isoferulic acid, an isomer of FA, also significantly inhibited the proliferation of human renal carcinoma A-498 cells, induced cleaved caspase-3 expression, and promoted the apoptosis of A-498 cells; moreover, isoferulic acid dose-dependently downregulated the expression of β-catenin and MYC proto-oncogene (c-Myc), inducing apoptosis [36]. Therefore, isoferulic acid is considered a potential candidate for the treatment of renal carcinoma.

Yue et al. [37] evaluated the potential effects of FA's nitrate compound FXS-3 on the proliferation and metastasis of lung cancer A549 cells. The results showed that FXS-3 can inhibit the activity of A549 cells by upregulating the Bax/Bcl-2 ratio mediated by the c-Jun N-terminal kinase (JNK) and extracellular signal-regulated kinase (ERK)/p38signaling pathways, which provides an important scientific basis for the development of FA derivative anticancer drugs. Nitric oxide has a wide range of potential applications in tumor therapy [38]. Zhang et al. [37] designed and synthesized an FA-nitric oxide donor conjugate. After this coupling was applied to A549 lung cancer cells, it was found that it could upregulate the expression levels of Bax and JNK by downregulating the expression of,Bcl-2, P38 and ERK, thus inhibiting the proliferation of A549 cells and inducing their apoptosis.

### Reactive oxygen species (ROS)

ROS are a group of short-lived and highly active oxygen-containing molecules that can induce DNA damage and genotoxic stress, as well as initiate oxidative stress-induced tumor cell death [39]. After treatment with FA, Cao et al. [40] observed an increase in ROS production and a decrease in superoxide dismutase activity and glutathione content in EC-1 and TE-4 oesophageal cancer cells. In addition, FA could promote the release of lactate dehydrogenase (LDH) and the activation of caspase-3 in oesophageal cancer cells, thus inducing cell apoptosis. Rosaria et al. [41] found that FA can activate the ERK1/2 pathway through the participation of ROS and play a proapoptotic role in human glioblastoma U-87 MG cells by reducing the expression levels of Bcl-2, ERK1/2, and c-Myc.

Zinc oxide nanoparticles are effective carriers for the targeted delivery of anticancer drugs to tumor cells [42]. Babu et al. [43] conjugated zinc oxide nanoparticles with FA (ZnONPs-FA) to act on hepatoma Huh-7 and HepG2 cells. The results showed that ZnONPs-FA could induce oxidative DNA damage and apoptosis by inducing ROS production. Therefore, ZnONPs-FA may be a promising drug for the treatment of liver cancer.

### Proliferating cell nuclear antigen (PCNA) pathway

**Fig. 1:**
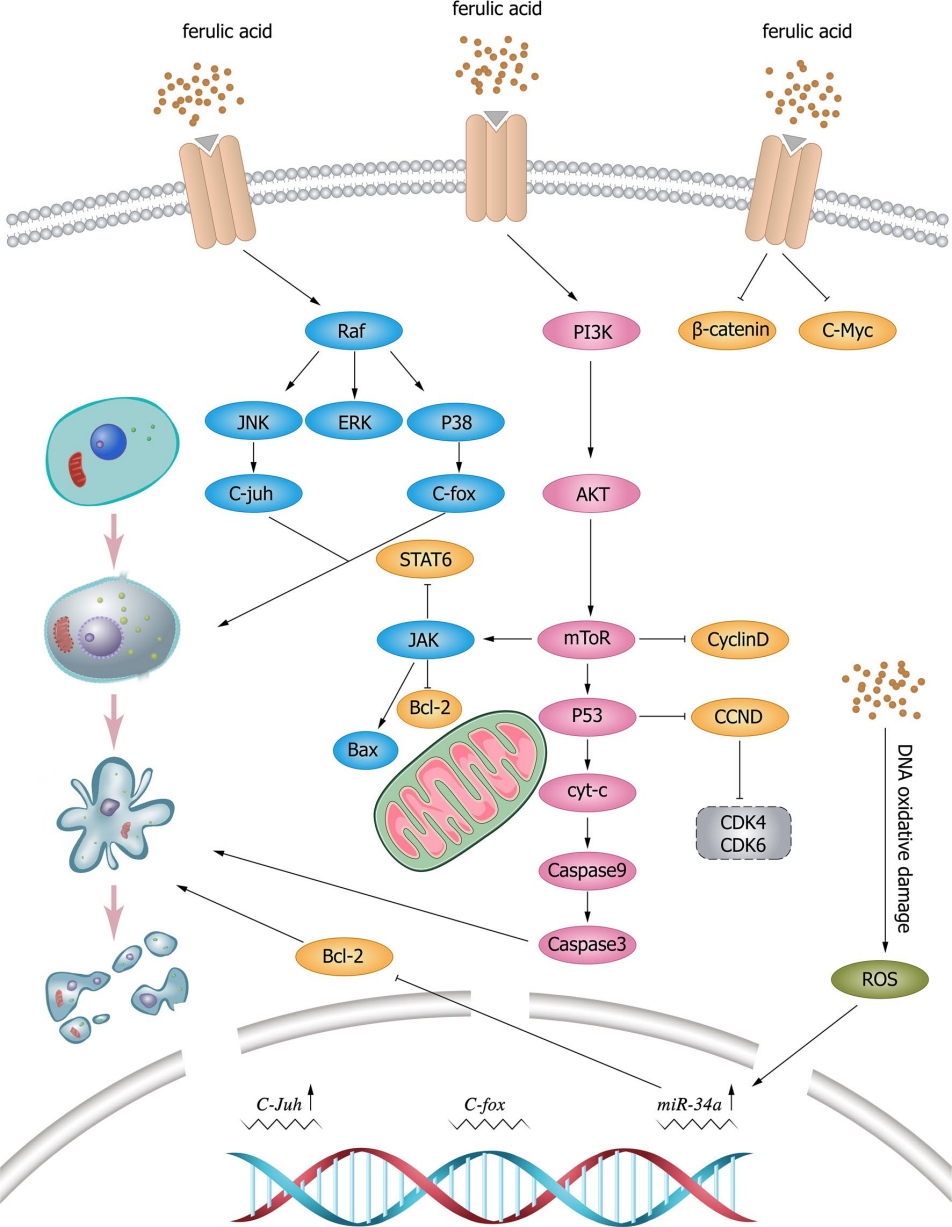
Mechanism of tumor cell apoptosis induced by FA and its derivatives. FA induces apoptosis by upregulating the expression of P53, reducing the expression of cyclin D1 and cyclin-dependent kinase (CDK) 4/6, upregulating Mir-43a, inhibiting the expression of Bcl-2, and activating the apoptosis pathway to promote cell apoptosis. FA causes DNA oxidative damage and apoptosis by inducing ROS production. *JNK* c-Jun N-terminal kinase, *ERK* extracellular signal-regulated kinase, *C-fox* forkhead box C, *STAT* signal transduction and transcriptional activator, *JAK* Janus kinase, *BCL-2* B-cell lymphoma-2, *Bax* Bcl-2-associated X, *PI3K* phosphatidylinositol 3 kinase, *AKT* protein kinase B, *mTOR* mammalian target of rapamycin, *P53* tumor protein 53, *Cyt-c* Cytochrome C, *Caspase* cysteinyl aspartate specific proteinase, *C-Myc* MYC proto-oncogene, *CCND* cyclin D, *CDK* cyclin-dependent kinases, *ROS* reactive oxygen species

PCNA is the core of many basic cellular processes, such as DNA replication, DNA damage, repair, and chromatin structure maintenance [44, 45]; it is also an excellent target for cancer therapy [46]. Arvind et al. [47] encapsulated FA and aspirin in a new chitosan-coated solid lipid nanoparticle (c-SLN) and observed the potential therapeutic effect of this drug delivery method. The results showed that the viability of MIA PaCa-2 and Panc-1 cells decreased significantly after c-SLN treatment. In in vivo studies of oral administration of c-SLN to a pancreatic cancer transplant mouse model, tumor growth was significantly inhibited compared with controls. In addition, immunohistochemical analysis showed significantly reduced expression of PCNA and MKI67 and increased expression of the apoptotic proteins p-RB, p21, and p-ERK1/2, indicating the proapoptotic effect of this regimen. The mechanisms by which FA induces apoptosis in cancer cells are shown in Fig. 1.

## Inhibition of tumor cell proliferation in response to FA

Cell proliferation is an important part of cell growth and differentiation [48], and active proliferation and metabolism are characteristics of tumor cells [49]. Unlike normal cells, cancer cells have the characteristics of infinite growth, transformation and metastasis and, therefore, are difficultly eliminated. Thus, inhibiting the proliferation of tumor cells is an important method of tumor therapy. In vitro and in vivo studies have shown that FA can effectively inhibit the proliferation of colorectal cancer, lung cancer and breast cancer cells in a variety of ways.

### Cell cycle blockade

The cell cycle is regulated by various factors [50], and impairment of its regulatory mechanism of the cell cycle can lead to uncontrolled growth of normal cells and transformation into tumor cells. Therefore, targeting cell cycle components may be an effective anticancer strategy. Wang et al. [51] showed that the survival rate of osteosarcoma 143B cells after FA treatment was significantly reduced. Compared with that in the control group, the proportion of G_0_/G_1_ phase cells in the FA treatment group was significantly increased, the expression levels of CDK2, CDK4, and CDK6 were decreased, and the ratio of Bax/Bcl-2 was increased. Further studies showed that FA can inhibit phosphatidylinositol 3 kinase (PI3K)/ protein kinase B (AKT) in a dose-dependent manner [52]. These results suggest that FA may inhibit the proliferation and induce the apoptosis of osteosarcoma cells by inhibiting the PI3K/Akt pathway. Canan et al. [53] observed that FA can inhibit cell proliferation by increasing the gene expression of TP53 and decreasing the gene expression of CDK2, CDK,4 and CDK6 in prostate cancer PC-3 cells, thus leading to cell cycle arrest in PC-3 cells. Luo et al. [54] found that FA can significantly reduce the number of S phase and G_2_/M phase cells in HCT116 and HT-29 colorectal cancer cells and increase the proportion of G1 phase cells in these two cancer cell lines, which reflects its antitumor cell proliferation ability.

Gao et al. [55] treated cervical cancer HeLa and CaSki cells with FA, and cell growth was significantly inhibited. Further studies showed that FA induced HeLa and CaSki cells to stay in the G_0_/G_1_ phase of the cell cycle in a dose-dependent manner, while FA induced the expression of cycle-related proteins such as p53 and p21 and decreased the expression levels of cyclin D1 and cyclin E. Anwar et al. [56] reported that FA could significantly reduce the proportion of cells in S phase, thus inhibiting the proliferation of the breast cancer cell line MDA-MB-231. However, in contrast to the above results, the proportion of cells in other phases of the cell cycle did not change significantly in this study, and the specific reasons remain unclear.

### Janus kinase/signal transduction and transcriptional activator (JAK/STAT)

The JAK/STAT3 signaling pathway is involved in almost all immune regulatory processes, including tumor cell recognition and tumor-driven immune escape [57]. Therefore, inhibitors targeting the JAK/STAT3 pathway may inhibit tumor cell growth and stimulate antitumor immunity. Studies have shown [58] that FA can effectively reduce the expression levels of the immune factors IL-4, platelet-derived growth factor (PDGF), and granulocyte–macrophage colony-stimulating factor(GM-CSF) as well as the phosphorylation level of JAK2/STAT6 in lung cancer A549 cells, suggesting that FA can inhibit the proliferation and metastasis of lung cancer cells by inhibiting the JAK2/STAT6 immune signaling pathway.

### Nuclear factor-kappa B-gene binding (NF-κB) pathway

Dysfunctional NF-κB activity is associated with inflammatory diseases and cancer [59]. The NF-κB-signaling pathway has long been used as a potential target for disease treatment because it activates antiapoptotic genes and downregulates the expression of proapoptotic factors to induce tumor cell growth. FA can effectively inhibit the activation of the NF-κB signaling pathway [60]. Hiroko et al. [61] showed that FA can reduce tyrosinase activity by directly binding to enzymes and inhibit the tyrosinase phosphorylation induced by casein kinase 2 (CK2) in B16 melanoma cells in a dose-dependent manner in vitro, thus inhibiting the activation of NF-κB.

### Overexpression of the pyrroline-5-carboxylate reductase 1 (PYCR1) pathway

Overexpression of PYCR1 is associated with the occurrence and development of cancer [62]. Yang et al. [63] found that FA could inhibit the proliferation of breast cancer MCF-7 and 4T1 cells in a dose-dependent manner, and further studies found a direct interaction between FA and PYCR1. PYCR1 can catalyze proline metabolism and synthesis in vivo through enzymatic reactions, which play a role in promoting tumor growth and proliferation. FA can target PYCR1 and inhibit its enzyme activity in a concentration-dependent manner.

### PI3K/AKT/mammalian target of rapamycin signaling pathway

**Fig. 2:**
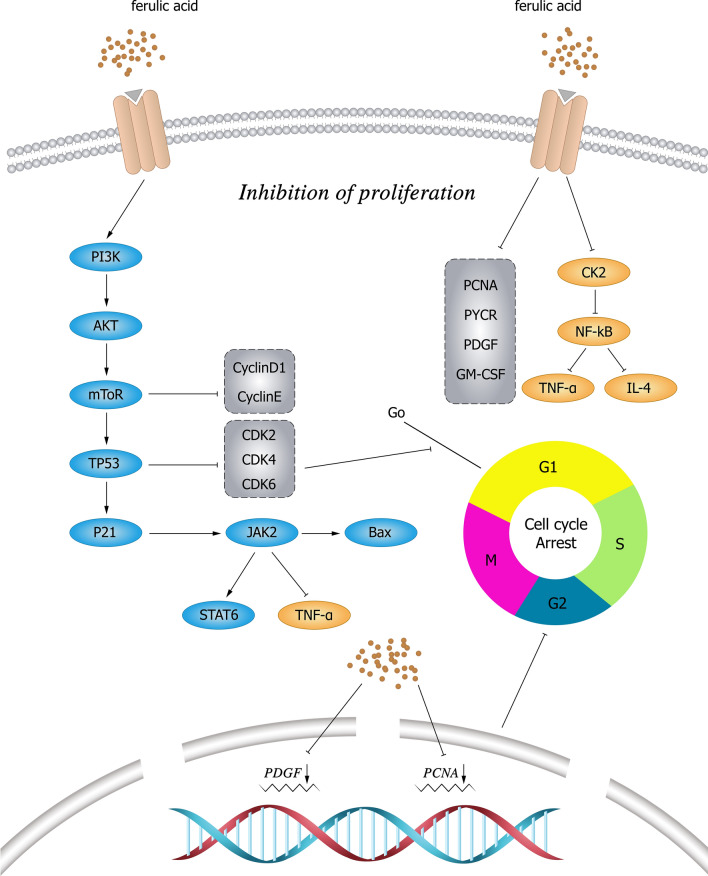
Mechanism of FA and its derivatives inhibiting tumor cell proliferation. FA can inhibit cell proliferation by increasing the gene expression of TP53 and decreasing the gene expression of CDK2, CDK4, and CDK6, thereby leading to cell cycle arrest. It also reduces the expression levels of IL-4, PDGF, and GM-CSF and the phosphorylation level of JAK2/STAT6 and inhibits the JAK2/STAT6 signaling pathway to suppress cell proliferation and metastasis. FA inhibits casein kinase 2 (CK2)-induced phosphorylation of tyrosinase, thereby inhibiting the activation of NF-κB. FA reduces the phosphorylation of Akt and PI3K and inhibits the activation of the PI3K/AKT pathway. *PI3K* phosphatidylinositol 3 kinase, *AKT* protein kinase B, *mTOR* mammalian target of rapamycin, *TP53* tumor protein 53, *CDK* cyclin-dependent kinases, *JAK* Janus kinase, *STAT* signal transduction and transcriptional activator, *TNF-α* tumor necrosis factor-α, *Bax* Bcl-2-associated X, *PCNA* proliferating cell nuclear antigen, *PDGF* platelet-derived growth factor, *GM-CSF* granulocyte–macrophage colony-stimulating factor, *NF-κB* nuclear factor-k-gene binding, *IL* interleukin

The PI3K/AKT/mTOR signaling pathway plays an important role in the regulation of cell proliferation, apoptosis, metabolism, and angiogenesis [64]. FA inhibits the activation of the PI3K/AKT pathway [65]. Luo et al. [66] found that in CaSki cells, phosphorylation of Akt and PI3K was reduced by FA in a concentration-dependent manner, leading to cytotoxicity and apoptosis of CaSki cells. Wu et al. [67] found that FA could significantly reduce the expression level of mTOR mRNA and Ki-67 protein in A549 lung cancer graft tissue, increase the expression of caspase-3 protein, and significantly inhibit the growth of tumors. The mechanisms by which FA inhibits tumor cell proliferation are shown in Fig. 2.

## Inhibition of tumor cell metastasis and invasion in response to FA

The process of tumor cells invading and metastasizing from the primary lesion to the surrounding tissues and then invading the surrounding tissues or even spreading throughout the whole body is called tumor invasion and metastasis. Cancer cell metastasis is an important cause of cancer death [68]. The migration and invasion abilities of tumor cells play an important role in the metastasis and recurrence of tumors. Inhibiting the invasion and metastasis of tumor cells can effectively interfere with the cancer process.

### Vascular endothelial growth factor (VEGF)

Antiangiogenic therapy is an effective means for the treatment of solid cancer [69]. VEGF is the main medium of angiogenesis, and the application of antiangiogenic agents targeting VEGF is an important strategy for the treatment of many cancers [70, 71]. Zhang et al. [72] found that FA could reduce the expression level of VEGF mRNA and protein in osteosarcoma SaOS-2 cells, and the expression level of CD34 protein in the metastatic tissue of nude mice treated with FA was significantly decreased. The results suggest that FA can reduce osteosarcoma's blood supply capacity by inhibiting the vascularization process to achieve antitumor effects. Riham et al. [73] prepared FA nanoparticle liposomes. In vitro*,* studies revealed that FA liposomes showed antiangiogenic potential by regulating the expression of cyclin D1 and VEGF after acting on colorectal cancer HCT116 and Caco2 cells. The results show that FA lipid nanoliposomes are an ideal system for the treatment of colorectal cancer and can be used as an effective measure to prevent metastasis.

### Fibroblast growth factor 1 (FGF1)

FGF1 plays an important role in tumor progression as a mediator leading to increased invasion and migration [74, 75]. Yang et al. [76] found that FA could inhibit endothelial cell proliferation, migration, and response to FGF1. In vivo*,* angiogenesis measurements showed that FA inhibited FGF1-induced angiogenesis of the rat aortic ring microvasculature. Further studies showed that FA inhibited FGF1-triggered fibroblast growth factor receptor 1 (FGFR1) and PI3K/Akt signaling [76]. These results suggest that FA is a novel FGFR1 inhibitor with potential antiangiogenic and anticancer activities.

### Epithelial–mesenchymal transition (EMT)

EMT is a complex biological transdifferentiation process that confers mesenchymal characteristics to epithelial cells [77]. EMT is considered an important factor in cancer invasion and metastasis. In the course of cancer, EMT is closely related to tumor occurrence, invasion, metastasis and treatment resistance [78]. Zhang et al. [79] found in a Transwell experiment that FA had a dose-dependent inhibitory effect on the migration of MDA-MB-231 breast cancer cells, and even low concentrations of FA could inhibit the migration of breast cancer cells. Further studies on protein and mRNA levels showed that E-cadherin significantly increased and vimentin, Snail, and Slug proteins decreased after FA treatment. These results confirm that FA can inhibit EMT and can inhibit tumor metastasis. Matrix metalloproteinases (MMPs) are closely related to angiogenesis, invasion, metastasis, and the avoidance of immune surveillance in the course of cancer, among which MMP1 and MMP9 are universally upregulated in almost all cancers [80]. Park et al. [81] showed that FA could inhibit the expression of MMP1 and MMP9 in melanoma B16F10 cells and had no cytotoxicity when the concentration was as high as 20 μM, showing its potential as a functional food. Yavuz et al. [82] found that the gene expression of MMP-2 and MMP-9, which are responsible for extracellular matrix degradation, was significantly reduced, TIMP1 expression was increased and invasion and migration were decreased in FA-treated TT human thyroid cancer cells compared to control TT human thyroid cancer cells.

### Carbonic anhydrase IX (CAIX) pathway

**Fig. 3:**
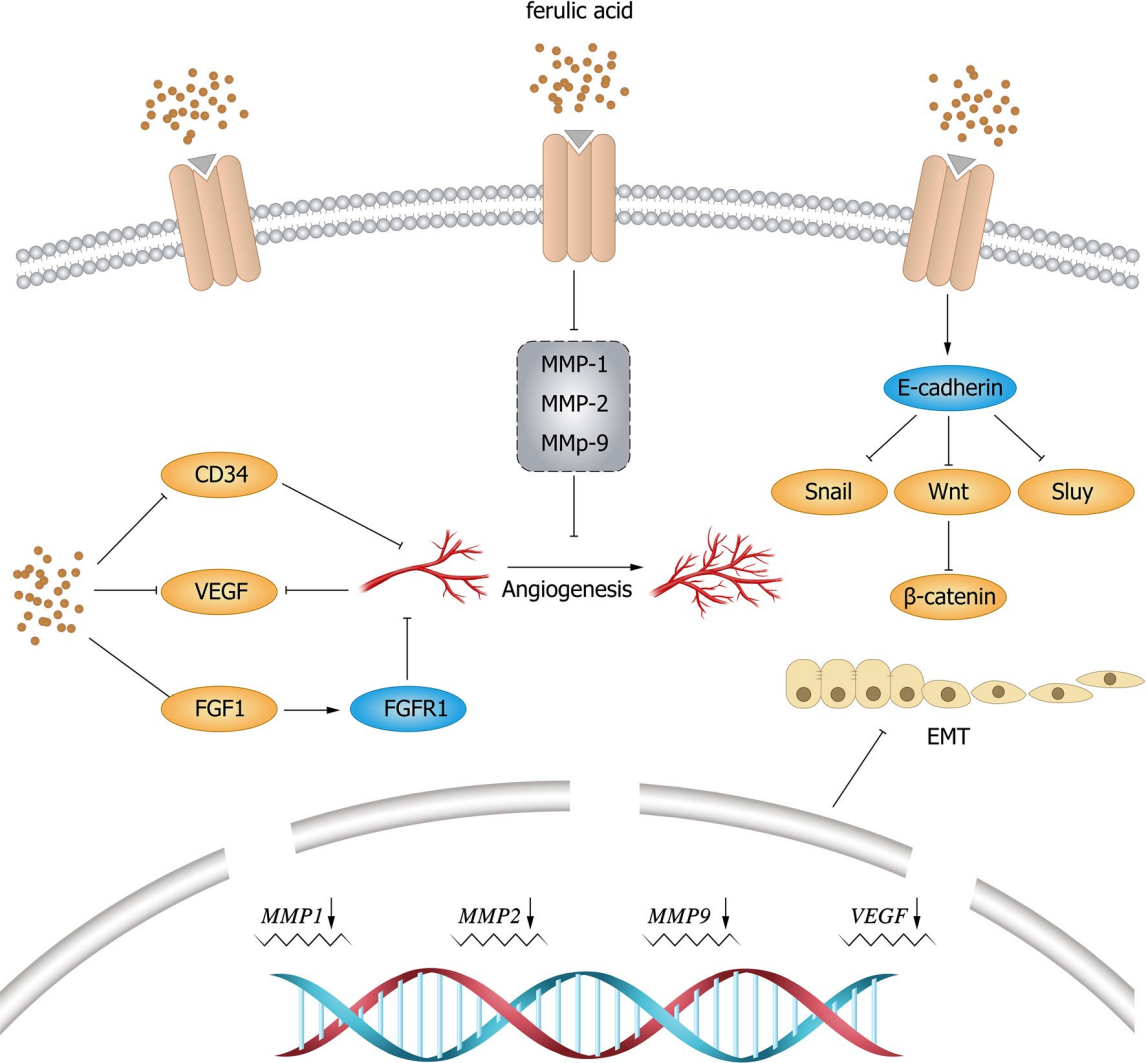
Mechanism of FA and its derivatives inhibiting tumor cell invasion and migration. FA inhibits cell invasion and migration by reducing the expression levels of VEGF mRNA and protein and CD34 protein. FA inhibits FGF1-induced angiogenesis and inhibits invasion and migration. FA can inhibit the expression of MMP1 and MMP9 in cells, inhibit EMT, and further suppress cell invasion and metastasis. *VEGF* vascular endothelial growth factor, *FGF* fibroblast growth factor, *FGFR* fibroblast growth factor receptor, *EMT* epithelial-mesenchymal transition, *MMP* matrix metalloproteinase

CAIX is a kind of transmembrane protease induced by a hypoxic microenvironment that belongs to the zinc metalloproteinase family; its high expression in the tumor cell membrane is caused by hypoxia. CAIX, which is induced and activated by hypoxia in the tumor process, is involved in the molecular mechanism of cancer cell invasion and metastasis and has been widely studied as a therapeutic target for cancer [83, 84]. Babita et al. [85] synthesized an FA-triazole compound, and the results showed that this compound could inhibit the activity of HT-29 and HepG2 cells in a concentration-dependent manner. CAIX expression in treated HT-29 and HepG2 cells was significantly reduced, and the toxicity to HEK293 cells of the embryonic kidney was small within the same drug concentration range. The results suggest that this compound has good anticancer properties and can selectively inhibit CAIX, indicating that the use of FA as a CAIX inhibitor has good potential as a targeted cancer therapy. The mechanism by which FA inhibits the invasion and metastasis of cancer cells is shown in Fig. 3.

## Enhanced sensitivity to radiotherapy/chemotherapy

The tolerance of tumor cells to chemoradiotherapy after a period of treatment is a serious problem. Commonly used chemotherapy drugs easily cause patient intolerance or tumor drug resistance, so it is urgent to develop new clinical drugs that have increased effectiveness and reduced toxicity.

FA is poor in water solubility, and its sodium salt, sodium ferulate, has high solubility and stable properties. It can be synthesized artificially, and after years of research and development, it has been widely used in the field of medicine. Resveratrol, a natural medicine, has a strong antitumor ability [86]. Yuuga et al. [87] found that novel derivatives of FA coupled with resveratrol could inhibit the proliferation of colon cancer HCT116 cells. It was further found that FA derivatives increased the mRNA level of the tumor suppressor p15, a CDK inhibitor. These results suggest that FA derivatives inhibit tumor cell proliferation by increasing p15 expression in HCT116 cells.

Subburayan et al. [88] reported that FA can produce free radicals, so FA treatment before radiotherapy could enhance the radiation effect of cancer cells. It was also confirmed by experiments that FA can increase ROS, lipid peroxidation markers, and DNA damage in HeLa and ME-80 cervical cancer cells, thereby reducing cell viability, survival rate, and antioxidant status and enhancing radiotherapy sensitivity. Das et al. [89] observed that the ROS levels in the treatment group were significantly higher than those in the control group after FA was used to pretreat lung cancer A549 and liver cancer HepG2 cells before radiotherapy. In this case, exposure to γ rays would further increase ROS levels and improve the radiosensitivity of cancer cells, indicating that the combination of FA and radiotherapy could activate the mitochondrial apoptotic pathway. In addition, compared with radiotherapy alone, combination therapy showed tumor regression, caspase-3 activation, and nuclear fragmentation in tumor tissues. In addition, FA pretreatment protected peripheral blood mononuclear cells (PBMCs) and normal lung fibroblasts (WI38) from radiation damage. In conclusion, FA preconditioning before radiotherapy provides an effective strategy to kill cancer cells and demonstrates the potential to improve radiotherapy efficacy.

Zheng et al. [90] synthesized stable PFA&DOX nanoparticles (NPs) with doxorubicin (DOX) from poly ferulic acid (PFA) chemically modified by FA and found that NPs could sustainably release active components under slightly acidic conditions in vitro, ensuring effective drug administration in the acidic tumor microenvironment. In vivo*,* antitumor studies showed that PFA nanocarriers can promote the accumulation of drugs at the tumor site to obtain better antitumor effects. In addition, PFA&DOX NPs reduced the toxicity of free DOX, thus improving the safety, suggesting that these drug-loaded NPs can provide a reference for future clinical antitumor drug development.

FA also inhibited P-glycoprotein (P-gp) transport in drug-resistant KB-Ch(R)-8-5 cells and significantly downregulated ABCB1 gene expression in a concentration-dependent manner. Cytotoxicity analysis showed that FA reduced paclitaxel resistance in KB-Ch(R)-8-5 cells and upregulated apoptosis signaling in paclitaxel-induced resistant cells, demonstrating its potential for chemical sensitization [91]. These results suggest that the downregulation of ABCB1 and subsequent apoptosis signal transduction may be responsible for the chemical sensitization potential of FA in P-gp-overexpressing cell lines. However, another study [92] pointed out that although FA can offset the ototoxicity of cisplatin to a certain extent, FA shows unnecessary tumor-protective effects, which may limit its clinical use. Therefore, how to ensure the safety of drug use while taking into account the antitumor efficacy is still a problem worth exploring.

## Reversing the drug resistance of tumor cells

Tumor cells can mutate under prolonged drug use, showing adaptability. Once tumor resistance occurs, the drug cannot exert its normal anticancer effect. Low intracellular drug concentrations are an important cause of drug resistance in tumor cells.

Hyaluronic acid nano gels can carry drugs well, promote their release in acidic environments, and increase intracellular drug concentrations [93]. CD44, a receptor of hyaluronic acid, has higher expression in many tumor cells than in normal tissues and is associated with the tumorigenicity, invasiveness, and lymphatic metastasis of tumor cells. Zhao et al. [94] found that hyaluronic acid-FA could effectively inhibit the growth of Lewis lung cancer tissue, and its inhibitory effect was comparable to that of cyclophosphamide, indicating that hyaluronic acid-FA targeted drugs could deliver drugs to cells with high CD44 expression through a receptor-mediated mechanism, thus achieving the purpose of enhancing drug efficacy.

Ganesan et al. [95] studied the regulatory effect of FA on P-gp-mediated multidrug resistance (MDR). The results showed that FA could enhance the cytotoxicity of alamycin and vincristine in KB-Ch(R)-8-5 cells overexpressing P-gp. In addition, FA combined with adriamycin significantly reduced the tumor volume of KB-Ch(R)-8-5 xenogeneic tumors compared with adriamycin alone. Previous studies showed that activation of NF-κB by the PI3K/AKT pathway may lead to increased transcription of MDR1 [96]. It is suggested that FA can reverse MDR by inhibiting the expression of P-gp through inhibition of the PI3K/Akt/NF-κB signaling pathway. Rajeshkumar et al. [97] prepared a nanopolymer of FA and paclitaxel (PTX) supported polyamidoamine (PAMAM) dendrimer coupled with arginine-glycine aspartic acid (RGD). The levels of proapoptotic factors such as caspase 3, caspase 9, p53, and Bax were increased, and the antiapoptotic factors were downregulated in the drug-resistant KB-CH(R)-8-5 cells treated with RGD-PAMAM-FP. The results showed that the polymer could overcome P-gp-mediated MDR. Trans FA could reduce the side effects of doxorubicin and cyclophosphamide, protect the normal function of the heart, liver, and bone, and restore frontal chemotherapy sensitivity in breast cancer model mice by reducing the level of P-gp [98].

Oxaliplatin is a commonly used chemotherapy drug for the treatment of colorectal cancer. Zhang et al. [99] found that FA can significantly improve the sensitivity of drug-resistant HCT116 colorectal cancer cells to oxaliplatin (OXA). The mechanism is that FA interferes with the expression of the MDR1 gene by blocking the catabolism of ganglioside acid (GM3). Neuraminidase 3 (NEU3) can catalyze the conversion of GM3 into ceramide trihexosides (Gb3). FA inhibits the activity of NEU3 and then reduces the levels of Gb3 and P-gp, thus improving the sensitivity of drug-resistant cells to OXA chemotherapy. These results suggest that FA can increase chemotherapy sensitivity by remodeling Neu3-mediated GM3 ganglioside metabolism and nay become a new drug to promote the efficacy of chemotherapy in colorectal cancer.

## Other antitumor effects

### Autophagy

Autophagy is the basis of cell component degradation and recycling. Autophagy is closely related to malignant transformation, tumor progression, and treatment [100]. FA treatment can enhance autophagy and increase the expression of autophagy-related proteins, resulting in the inhibition of mTOR signal transduction [101].

Wang et al. [102] found that FA could increase the levels of apoptosis and the autophagy biomarkers PINK-1 and Parkin in hepatocellular carcinoma HepG2 cells, suggesting that FA could inhibit the proliferation of HepG2 cells and increase their apoptosis and autophagy. Autophagy is the core target of FA in the treatment of osteosarcoma. Pang et al. [103] identified activators of signal transducer and activator of transcription 3 (STAT3), mitogen-activated protein kinase 1 (MAPK1), and phosphoinositin-3 kinase regulatory subunit 1 (PIK3R1) as pharmacological targets of FA in the treatment of osteosarcoma through molecular docking analysis. These findings from bioinformatics analysis demonstrate the potential therapeutic role of FA in the treatment of osteosarcoma. Claudia et al. [104] synthesized a novel compound, FA tributyltin, based on FA and evaluated its effects on colon cancer cells. The results showed that the compound could reduce the activity of colon cancer HCT116, HT-29, and Caco-2 cells. Further studies showed that the decrease in cell viability induced by this compound was associated with G_2_/M cell cycle arrest, a process that hardly involved changes in apoptosis or necrotizing apoptotic markers. Autophagy vacuoles and increased LC3-II and p62 autophagy proteins were observed after treatment with this compound, suggesting that the process of cell death is triggered by autophagy. These results suggest that FA derivatives are promising antitumor agents that can trigger autophagy, which may be very important in the case of resistance to the classic apoptosis process.

However, autophagy can be a double-edged sword in different stages of cancer development. In early tumorigenesis, autophagy, as a survival pathway and quality control mechanism, can prevent tumorigenesis and inhibit cancer progression. Once the tumor progresses to an advanced stage, autophagy will instead contribute to the survival and growth of the tumor and promote the aggressiveness of cancer by promoting metastasis [105]. Autophagy plays roles in both tumor inhibition and promotion, so the exact mechanism of autophagy in cancer remains to be further studied.

### Chemical prophylaxis

Cancer chemoprophylaxis refers to the strategy of using natural or synthetic chemicals to prevent, reverse or slow down the development of cancer, which is a hot research field in cancer prevention. FA can prevent buccal pouch carcinogenesis induced by 7,12-dimethylbenzene [a] anthracene (DMBA) in hamsters. Oral FA can significantly reduce the incidence, volume, and weight of tumors in hamsters, suggesting that FA has an effective chemopreventive effect on DMBA-induced buccal pouch carcinoma in hamsters [106].

The occurrence of skin cancer is associated with ultraviolet-b radiation (UVB) exposure [107]. In a study by Amboth [108], FA significantly reduced the incidence, volume, and weight of UVB-induced tumors in the skin of mice compared to control animals. It was also observed that FA therapy could reverse the chronic oxidative damage of UVB-induced skin tumors in mice while regulating the expression of VEGF, inducible nitric oxide synthase (iNOS), tumor necrosis factor-α (TNF-α), and IL-6. FA treatment also modulated the expression of mutated p53, Bcl-2, and Bax in UVB-induced mouse skin tumors. The results showed that FA had a potential inhibitory effect on UVB-induced carcinogenesis in albino mice. In addition, FA protects against the cytotoxicity induced by cyclophosphamide in neuroblastoma SH-SY5Y cells by reducing lipid peroxidation levels [109].

### Aerobic glycolysis

Metabolic changes are the hallmark of cancer, and reprogramming of energy metabolism is a common phenomenon in tumor progression. The change in aerobic glycolysis during the energy metabolism of cancer cells is a signature feature, also known as the Warburg effect [110]. Long noncoding RNAs (lncRNAs) regulate energy metabolism in cancer [111]. Cui et al. [112] found that the proliferation of HCT116 and HT-29 cells was inhibited after FA treatment. Further studies showed that FA could inhibit the expression of PKM2 and block aerobic glycolysis. Since PKM2 was positively correlated with lncRNA 495810 expression in this study, it was speculated that FA might inhibit aerobic glycolysis through lncRNA 495810/PKM2 signal transduction. The antitumor effects of FA and its derivatives are shown in Table 1.

**Table 1:** The antitumor effects of ferulic acid and its derivatives

Mechanism	Cell/tissue type	Dose/concentration	Target	References
Apoptosis	SGC-7901	5, 7.5, and 10 mg/ml	p53	[28]
	MIA PaCa-2	500 μM/ml	p53, CCND1 and CDK4/6	[29]
	HT-29	25 μg/ml	p53, caspase-9 and caspase-3	[31]
	Cervical neoplasms	50 mg/kg	miR-34a and Bcl-2	[34]
	MGC-803	5, 7.5, and 10 mg/ml	Bax/Bcl-2	[35]
	A-498	50 μmol/l	β-catenin and c-Myc	[36]
	A549	50 mmol/l	JNK and ERK/p38	[37]
	A549	3.7 μM	Bax/Bcl-2, JNK and ERK	[37]
	EC-1 and TE-4	20, 40 and 60 μM	ROS and caspase-3	[40]
	U-87 MG	36 µM	ERK1/2 and c-Myc	[41]
	Huh-7 and HepG2	4.1 µg/ml	ROS	[43]
	MIA PaCa-2 and Panc-1	200 µM	PCNA, MKI67 and p-RB	[47]
Proliferation	143B	100 and 200 μmol/l	CDK2, CDK4 and CDK6	[51]
	143B and MG63	100 and 150 μM	PI3K/Akt	[52]
	PC-3	1 and 2 mM	TP53	[53]
	Hela and CaSki	2.0 mM	p53 and p21	[55]
	MDA-MB-231	250, 350 and 450 mM		[56]
	A549	2, 4 and 8 mg/ml	JAK2/STAT6, PDGF and GM-CSF	[58]
	B16	100 µM	CK2 and NF-κB	[61]
	MCF-7 and 4T1		PYCR1	[63]
	CaSki	16, 18, 20 and 25 μM	PI3K/AKT	[66]
	A549	25, 50 and 100 mg/l	mTOR and Ki-67	[67]
Metastasis	SaOS-2	10, 20 and 40 μM	VEGF and CD34	[72]
	colon cancer	100 mg/kg	Cyclin D1 and VEGF	[73]
	B16F10	1 µM	FGFR1 and PI3K/Akt	[76]
	MDA-MB-231	12.5, 25 and 50 µM	E-cadherin, Snail and Slug	[79]
	B16F10	20 μM	MMP1 and MMP9	[81]
	TT	150 μM	MMP2, MMP9 and TIMP1	[82]
	HT29 and HepG2	8.44 and 11.22 μM	CAIX	[84]
Enhanced sensitivity to chemotherapy	HCT116	5.86 μM	p15, CDK	[87]
	HeLa and ME-80	20, 30 and 40 μg/ml	ROS	[88]
	A549 and HepG2	300 μM	ROS and caspase-3	[89]
	KB-Ch(R)-8–5	30 μM	P-gp and ABCB1	[91]
Reversed drug resistance of tumour cells	Lewis lung cancer	10 mg/kg	CD44	[94]
	KB-Ch(R)-8–5	10, 20 and 30 μM	P-gp	[95]
	KB-Ch(R)-8–5	10 μg/ml	caspase-3, caspase-9, p53 and Bax	[97]
	HCT-116	0.724 mg/ml	GM3, MDR1 and NEU3	[99]
Autophagy	HepG2	40–160 μg/ml	PINK-1 and Parkin	[102]
	osteosarcoma		STAT3, MAPK1 and PIK3R1	[103]
	HCT116, HT-29 and Caco-2	10 mM	LC3-II and p62	[104]
Aerobic glycolysis	HCT116 and HT-29	60, 120 and 180 µg/ml	lncRNA 495810/PKM2	[112]

In addition, Priya et al. [113] prepared polyethylene oxide nanofibres by electrospinning technology, and these nanofibres could continuously release effective active substances within 24 h of administration and showed a certain apoptotic effect on breast cancer MCF-7 cells. Tetrahydroisoquinoline plays an important role in the field of pharmaceutical chemistry due to its extensive pharmacological properties, making it an important scaffold for the design of anticancer drugs and a hot topic in current research [114]. Wang et al. [115] synthesized tetrahydroisoquinoline FA derivatives, which were detected by the SRB method to have certain in vitro antitumor activity in colon cancer HT-29 cells and breast cancer MCF-7 cells. Eldin et al. [116] grafted FA onto oligosaccharides, which were then self-assembled into particles. In vitro experiments showed that FA-grafted oligosaccharides have high anticancer activity against human colon cancer HT-29 and LoVo cells, suggesting that FA-grafted oligosaccharides are a good candidate material for relieving colorectal cancer. These results all indicated that FA-related derivatives had excellent antitumor functions but failed to elucidate the specific antitumor mechanism.

## Existing problems and prospects

As a phenolic substance widely present in plant cell walls, FA has been shown to have a broad-spectrum antitumor effect, inhibiting lung cancer, liver cancer, breast cancer, cervical cancer, colorectal cancer, and other related cancers. The anticancer effects of FA are mainly achieved by inhibiting the proliferation of tumor cells, inducing the apoptosis of tumor cells, inhibiting the invasion and migration of tumor cells, and enhancing the efficacy of chemoradiotherapy drugs. In addition, FA can play a role by regulating immune function [19, 117], inducing autophagy, and inhibiting the drug resistance of tumor cells. With the continuous development and improvement of experimental technology, data mining, bioinformatics, and other technologies, the antitumor effect of FA has been fully confirmed in experiments, and its pharmacodynamic mechanism has also been elaborated at multiple levels.

However, there are still some obstacles to the application of FA components as drugs in clinical treatment. On the one hand, FA has poor solubility in water and a poor ability to pass through biological barriers [118]; therefore, the extent to which it is metabolized in vivo after oral administration is largely unknown, and its absorption status in vivo and concentration in the target site is not easy to determine. Nanocarriers can overcome the restriction of drug action by body barriers (such as the blood–brain barrier and blood–eye barrier) and can reduce drug doses, improve drug availability and reduce side effects after modification by targeted groups. However, there is a lack of research on improving the bioavailability of monomer components and targeting them specifically to tumor tissue through chemical modification. On the other hand, we noted that there were significant differences in the doses used in the antitumor studies of FA, which were still in the exploratory stage and failed to form a unified standard. The above problems may be the reason why FA and its related components have not been developed into clinical drugs. Therefore, in future research, it is necessary to develop a carrier that can selectively deliver drugs to certain parts of the body, identify mutant cells, control the release of drugs, determine the safe and effective dose of FA, and develop a more effective drug regimen to serve cancer patients more efficiently. This still requires further experimental exploration and research.

## Data Availability

Not applicable.

## Abbreviations

AKT: Protein kinase B
Bax: Bcl-2-associated X
Bcl-2: B-cell lymphoma-2
CAIX: Carbonic anhydrase IX
Caspase: Cysteinyl aspartate specific proteinase
CDK: Cyclin-dependent kinases
CK2: Casein kinase 2
CRC: Colorectal cancer
C-fox: Forkhead box C
C-Myc: MYC proto-oncogene
Cyt-C: Cytochrome C
c-SLN: Chitosan-coated solid lipid nanoparticle
DMBA: 7, 12-Dimethylbenzene [a] anthracene
DOX: Doxorubicin
EMT: Epithelial-mesenchymal transition
ERK: Extracellular signal-regulated kinase
FA: Ferulic acid
FFA: FA-loaded solid lipid nanoparticles and folate
FGF1: Fibroblast growth factor 1
FGFR1: Fibroblast growth factor receptor 1
Gb3: Ceramide trihexanides
GM3: Ganglioside acid
GM-CSF: Granulocyte–macrophage colony-stimulating factor
iNOS: Inducible nitric oxide synthase
JAK: Janus kinase
JNK: C-Jun N-terminal kinase
IL: Interleukin
lncRNA: Long noncoding RNA
MAPK1: Mitogen-activated protein kinase 1
MDR: Multidrug resistance
MMP: Matrix metalloproteinase
mTOR: Mammalian target of rapamycin
NEU3: Neuraminidase 3
NF-κB: Nuclear factor-k-gene binding
NPs: Nanoparticles
OXA: Oxaliplatin
PCNA: Proliferating cell nuclear antigen
PDGF: Platelet-derived growth factor
PFA: Poly ferulic acid
P-gp: P-glycoprotein
PIK3R1: Phosphoinositin-3 kinase regulatory subunit 1
PI3K: Phosphatidylinositol 3 kinase
PTX: Paclitaxel
PYCR1: Pyrroline-5-carboxylate reductase 1
P53: Tumor protein 53
ROS: Reactive oxygen species
STAT: Signal transduction and transcriptional activator
STAT3: Signal transducer and activator of transcription 3
TIMP1: Tissue inhibitors of metalloproteinase 1
TNF-α: Tumor necrosis factor-α
UVB: Ultraviolet-b radiation
VEGF: Vascular endothelial growth factor
ZnONPs-FA: Zinc oxide nanoparticles with FA
